# Chinese medical students’ disposition for critical thinking: a mixed methods exploration

**DOI:** 10.1186/s12909-021-02801-w

**Published:** 2021-07-16

**Authors:** Lei Huang, Angela Pei-Chen Fan, Na Su, Jessica Thai, Russell Olive Kosik, Xudong Zhao

**Affiliations:** 1 Tongji Hospital, School of Medicine, Tongji University, Shanghai, China; 2 School of Medicine, National Yang-Ming University, Taipei, Taiwan; 3 School of Medicine, Tongji University, Shanghai, China; 4 Department of Psychiatry, University of New Mexico, Albuquerque, NM USA; 5 Shanghai Pudong New Area Mental Health Center, School of Medicine, Tongji University, Shanghai, China

**Keywords:** Critical thinking disposition, CTDI-CV, Chinese medical students

## Abstract

**Background:**

Critical thinking (CT) is an essential competency for medical students. This study’s aim was to evaluate Chinese medical students’ disposition for CT and to explore the impact of current trends in medical education on students’ CT development.

**Methods:**

We used multistage stratified cluster sampling to recruit a total of 1241 medical students among five different years of training and from three medical institutions in China. The Critical Thinking Disposition Inventory-Chinese Version (CTDI-CV) and self-reported information were used to collect cross-sectional data. Based on the data from the CTDI-CV, 112 medical students in clinical course training from a single institution continued one-year follow-up. Their one-year CTDI-CV score changes were collected regarding various medical education variables.

**Results:**

The mean CTDI-CV score of the 1241 medical students was 287.04 with 729 (58.7%) students receiving a score of 280 or higher. There were statistically significant differences in schools attended(F = 3.84, *P* < 0.05), year of school attended(F = 10.32, *P* < 0.001), GPA(F = 6.32, *P* < 0.01), weekly time spent learning after class(F = 14.14, *P* < 0.001), attitude toward medicine(F = 28.93, *P* < 0.001), desire to be a doctor after graduation(t = − 3.35, *P* < 0.001), familiarity with CT(F = 20.40, *P* < 0.001), and perception of importance of CT(F = 22.25, *P* < 0.001). The participants scored the highest on the CTDI-CV subscales of “inquisitiveness” and the lowest on “truth seeking.” The 112 students in the longitudinal study had significantly lower total CT scores after one academic year follow-up.

**Conclusions:**

Chinese medical students generally exhibited positive CT dispositions. The cross-sectional survey and one-year longitudinal study indicated that students’ CT disposition diminished as they progressed through traditional medical training. Our study contributes to understanding the status of Chinese medical education of and influential factors on medical students’ CT disposition.

## Study descriptor

This study used both cross-sectional exploratory and longitudinal study designs.

## Background

Critical thinking (CT) is a philosophical concept, which involves individual characteristics, personality traits, and habits of the mind. It is a pervasive and self-rectifying human phenomenon and refers to dispositions and skills which reveal what is authentic, what to believe, why it is, and how it happens [1]. Medicine is universally acknowledged as a challenging profession due to the sacredness of human life and the complexity and uncertainty of the human body. CT is essential in helping medical students manage complex health situations and solve clinical problems effectively by sound decision-making. Previous studies have shown positive correlations among CT and clinical competency [2, 3], academic success [4], and research skills [5]. Therefore, the Institute for International Medical Education (IIME) has listed “critical thinking and research” as one of the seven essential competencies that a medical graduate should possess as part of the Global Minimum Essential Requirements (GMER) [6]. The Ministry of Education in the People’s Republic of China has also stated that “scientific attitude, innovation, and critical thinking” are essential requirements for Chinese medical graduates [7].

The importance of CT in medical education has been of wide concern in China. Studies have suggested that Chinese medical students exhibit a positive disposition towards CT [8–11]. However, CT scores were lower than that of health professional students from western countries [12]. Educational factors including curricular model, teaching methods, and evaluation systems were associated with medical students’ CT disposition [9, 11, 13]. The discipline-based curricular, which is widely implemented in China, may not sufficiently encourage development of Chinese medical students’ disposition for CT. This curriculum has isolated phases (theory, clerkship, and internship), limited faculty-student interaction, and a solely knowledge-based evaluation for the discipline-based curricular [14]. As a result, medical educators are becoming increasingly convinced that this model no longer meets the needs of the modern health-care system in China [15, 16].

To our knowledge, the majority of previous studies assessing Chinese medical students’ CT disposition did not include representative samples of study participants [9–11, 17]. The impacts of Chinese medical education on medical students’ CT via longitudinal study design was also limited [18]. As a result, this study was conducted to evaluate medical student CT disposition among different medical institutions of China and to explore the impact of medical education on students’ CT disposition via a one-year clinical course follow-up.

## Methods

### Participants

One thousand two hundred forty-one medical students were recruited by multistage stratified cluster sampling for a cross-sectional survey. In the first stage of the study, three Chinese medical institutions were selected, each of which was classified into a different medical school class to increase sample representation. The Ministry of Education categorizes Chinese medical schools into three classes. The first class of medical schools includes recipients of both the “985” and “211” grants (Tongji University School of Medicine, Shanghai in this study). The second class includes schools that have only received the “211” grant (Medical College of Soochow University, Jiangsu province). The third class includes schools not supported by national grants (Gannan Medical University, Jiangxi province). The “985” grant is the People’s Republic of China’s government- sponsored initiative to upgrade 30 selected research universities to internationally renowned status. The “211” grant was initiated by the Chinese government in the 1990s with the specific goal of increasing the implementation of pro-higher education policies [19]. In the second stage, two classes were randomly selected from each year of school (first through fifth year) in each institution. Approximately 100 students were chosen from each year within each institution. In the third stage, all students in the selected class were invited to participate in the study. Ultimately, 1521 medical students were invited to participate in this study and 280 students were excluded due to the incomplete data. The response rate was 81.6%. In this study, 615 (49.6%) participants were male and 626 (50.4%) were female. Students ranged from age 18 to 27 years old (mean = 22.04, SD = 1.75). Medical students were divided into three stages by school year. 518(41.7%) students were in basic medical science courses in their first to second years. 545(43.9%) students were in clinical courses in their third to fourth years, and 178(14.3%) students were in their internships in their fifth year. After the cross-sectional survey, 112 medical students from the clinical course stage at Tongji University School of Medicine were selected to participate in a one-year longitudinal study by convenience sampling. Eighty-one of them were third-year students, and 31 were fourth-year students.

### Instruments

#### Chinese critical thinking disposition inventory (CTDI-CV)

The CTDI-CV was translated and modified by Peng [20] based on Facione’s “California Critical Thinking Dispositions Inventory” (CCTDI) [21]. It is a standardized 70-item multiple choice test that examines each of the seven categories of CT disposition, including “truth seeking,” “open-mindedness,” “analyticity,” “systematicity,” “self-confidence,” “inquisitiveness,” and “cognitive maturity.” The overall Content Validity Index (CVI) of this test was 0.89, and the CVI of the test’s subscales ranged from 0.6 to 1. The overall alpha was 0.90, and the subscales alphas ranged from 0.54 to 0.77 [20]. In this study, the Cronbach’s Alpha Score was 0.841. Higher scores indicated stronger CT dispositions. Total scores over 280 and subscales over 40 were considered as positive attitudes towards critical thinking disposition [21].

#### Self-reported information

Personal information was collected, including (1) demographic factors: gender and age (2) academic factors: “school,” “year of school attended,” “GPA,” (with answers “above average meaning between 3.5-5.0,” “average meaning between 2.5-3.5,” “below average meaning less than 2.5,”), “weekly time spent learning after class” (3) attitude factors: “attitude towards medicine,” “desire to be a doctor after graduation,” “familiarity with CT,” and “perception of importance of CT.”

### Procedure

A questionnaire was organized with CTDI-CV and self-reported questions. A pilot study was conducted with 20 medical students at Tongji University School of Medicine before the official study and questionnaire. Both the pilot and official questionnaires consisted of CTDI-CV and self-reported questions. After the pilot study, an official questionnaire was organized and modified based on student feedback. The official questionnaires and consent forms were given to study participants via staff members from students’ respective medical education offices. Study participants were asked to return completed surveys in 2 weeks via a sealed return envelope for confidentiality. Participants were reminded 3 days before the 2-week deadline to complete their questionnaires. After questionnaire completion and collection, responses were analyzed and transformed via SPSS. Questionnaire answers left partially or fully uncompleted were regarded as incomplete answers or non-responses to answers. If missing data was greater than 5%, the questionnaire was excluded. Data analysis was guided by the statistician at our institution. After the cross-sectional survey, the group of 112 medical students at Tongji University School of Medicine completed the CTDI-CV at both the beginning and end of the following academic year. The project was approved by the Ethics Review Committee of Tongji Hospital of Tongji University (Registration Number K-2014-020), Medical College of Soochow University (Registration Number SUDA20210122H02), and Gannan Medical University (Registration Number 2014468).

### Statistical analyses

SPSS version 19.0 was used to conduct the following tests where relevant: independent T-test, one-way ANOVA, paired T-test, and McNemar test. A *p-*value of < 0.05 was statistically significant.

## Results

### Critical thinking disposition for medical students

The CTDI-CV scores ranged from 205 to 383, with a mean score of 287.04 (SD = 29.85). A score higher than the index score of 280 indicated a positive disposition. Chinese medical students in this sample, on average, displayed a positive disposition for CT. Almost 60 % of the students (*n* = 729, 58.7%) received a score of 280 or higher. In terms of sub-scale scores, the students scored the highest on “inquisitiveness” (index score 40, mean = 44.50 with SD = 6.13) and the lowest on “truth seeking” (index score 40, mean = 37.00 with SD = 6.21). When comparing medical school specific mean scores, Tongji University School of Medicine students received the highest CTDI-CV mean score (290.44) (Table 1).

**Table 1:** Mean scores of total and sub-scales of CTDI-CV

Dimensions	Gannan Medical University(***n*** = 429)	Medical College of Soochow University(***n*** = 413)	Tongji University School of Medicine(***n*** = 399)	Combined(***n*** = 1241)	
Subscales	Scores (Mean ± SD)	Scores (Mean ± SD)	Scores (Mean ± SD)	Scores (Mean ± SD)	Positive Attitude N (%)
Truth seeking	36.53 ± 6.30	37.21 ± 6.06	37.29 ± 6.25	37.00 ± 6.21	425(34.3)
Open-mindedness	40.39 ± 5.64	40.94 ± 5.13	41.74 ± 5.48	41.01 ± 5.45	754(60.8)
Analyticity	42.99 ± 5.67	43.38 ± 5.24	44.51 ± 6.01	43.61 ± 5.67	931(75.0)
Systematicity	38.56 ± 6.23	38.59 ± 5.40	39.42 ± 6.51	38.85 ± 6.07	533(43.0)
Self-confidence	41.17 ± 5.57	40.79 ± 5.52	42.15 ± 6.47	41.36 ± 5.88	753(60.7)
Inquisitiveness	45.26 ± 5.99	43.98 ± 6.11	44.46 ± 6.24	44.58 ± 6.13	981(79.1)
Cognitive maturity	40.55 ± 6.45	40.52 ± 6.11	40.88 ± 6.30	40.65 ± 6.29	757(61.0)
**Total Score**	285.44 ± 30.30	285.41 ± 27.61	290.44 ± 31.33	287.04 ± 29.85	729(58.7)

### Comparison of CTDI-CV Total scores with self-reported variables of medical students

There were significant differences among CTDI-CV total scores and variables defined in this study, including CT disposition among schools (F = 3.84, *P* < 0.05), years of school attended (F = 10.32, *P* < 0.001), GPA (F = 6.32, *P* < 0.01), weekly time spent learning after class (F = 14.14, *P* < 0.001), attitude towards medicine (F = 28.93, *P* < 0.001), familiarity with CT (F = 20.40, *P* < 0.001), and perception of importance of CT (F = 22.25, *P* < 0.001). Notably, “years of school attended” was negatively associated with CTDI-CV scores. Also of note was that medical students with “average GPA” had the lowest CTDI-CV scores vs. those with “above average GPA” who had the highest scores. Furthermore, an independent t-test indicated that the total CTDI-CV scores of students who wished to become medical doctors after graduation was significantly higher than those who did not (t = − 3.35, *P* < 0.001) (Table 2).

**Table 2:** Comparison of CTDI-CV total score based on self-reported variables

Demographics	N	%	Total Score (Mean ± SD)	Test	***p***
**School**					
Gannan Medical University	429	34.6	285.44 ± 30.30	F = 3.84	*p* < 0.05
Medical College of Soochow University	413	33.3	285.41 ± 27.61		
Tongji University School of Medicine	399	32.2	290.44 ± 31.34		
**Year of school attended**					
Basic sciences course (1st and 2nd)	518	41.7	290.80 ± 28.38	F = 10.32	*p* < 0.001
Clinical course (3rd and 4th)	545	43.9	285.92 ± 30.06		
Internship (5th)	178	14.3	279.51 ± 31.77		
**GPA**					
Above average	914	73.7	288.74 ± 29.72	F = 6.32	*p* < 0.01
Average	257	20.7	281.31 ± 29.00		
Below average	70	5.6	285.88 ± 32.29		
**Weekly time spent learning after class**					
> 10 h	466	37.6	291.25 ± 31.05	F = 14.14	*p* < 0.001
5 ~ 10 h	375	30.2	288.48 ± 27.46		
0 ~ 5 h	400	32.2	280.79 ± 29.61		
**Attitude towards medicine**					
Interest	794	64.0	291.66 ± 29.55	F = 28.93	*p* < 0.001
Ambivalent	387	31.2	279.74 ± 27.81		
No interest	60	4.8	273.03 ± 33.05		
**Desire to be a doctor after graduation**					
Yes	1136	91.5	287.90 ± 29.46	t = −3.35	*p* < 0.001
No	105	8.5	277.75 ± 32.49		
**Familiarity with CT**					
Understands	559	45.0	292.44 ± 31.14	F = 20.40	*p* < 0.001
Heard but does not understand	575	46.3	283.85 ± 27.15		
Never heard	107	8.6	275.97 ± 31.48		
**Perception of importance of CT**					
Important	910	73.3	290.28 ± 29.18	F = 22.25	*p* < 0.001
Ambivalent	282	22.7	279.26 ± 29.37		
Not important	49	4.0	271.58 ± 32.32		

Note: F: Ronald fisher value; t:hypothesis test value

### CTDI-CV Total score change after one-year of clinical courses

The group of 112 students who participated in the one-year longitudinal study exhibited a significant decline in total CTDI-CV scores as well as a decline in most subscales besides “systematicity.” Their mean total score dropped from 318.99 to 304.13. Statistically significant differences were found among total scores as well as the following subscales: “truth seeking,” “open-mindedness,” “analyticity,” “inquisitiveness,” and “cognitive maturity” before and after 1 year (*p* < 0.001) (Table 3). The percentage of medical students with a positive disposition for CT dropped from 73.21 to 66.96%. Furthermore, this drop was statistically significant by the McNemar test (*p* < 0.05) (Table 4).

**Table 3:** Comparison of CTDI-CV total score prior to and following one academic year

Dimensions	Prior to (Mean ± SD)	Following (Mean ± SD)	Pair t-Test	***p***
**Subscales Scores**				
Truth seeking	41.44 ± 3.24	39.26 ± 3.63	*t* = 11.86	*p* < 0.001
Open-mindedness	49.26 ± 4.44	44.94 ± 9.03	*t* = 11.91	*p* < 0.001
Analyticity	49.26 ± 4.20	44.47 ± 1.67	*t* = 15.14	*p* < 0.001
Systematicity	42.61 ± 3.45	43.09 ± 3.35	*t* = −1.28	*p* = 0.203
Self-confidence	46.02 ± 6.19	45.92 ± 8.47	*t* = 0.61	*p* = 0.546
Inquisitiveness	46.29 ± 2.41	45.76 ± 3.48	*t* = 7.35	*p* < 0.001
Cognitive maturity	44.10 ± 3.49	40.69 ± 7.26	*t* = 9.97	*p* < 0.001
**Total Score**	318.99 ± 14.91	304.13 ± 33.63	*t* = 10.20	*p* < 0.001

**Table 4:** Comparison of CTDI-CV Classification Prior to and Following One Academic Year

Classification of CT	Prior to		Following		Chi-square	***p***
	N	%	N	%		
Positive Attitude	82	73.2	75	67.0	*χ*^*2*^ *=* 19.29	*p* < 0.01
Negative Attitude	30	26.8	37	33.0		

## Discussion

CT is a prerequisite for safe practices in medicine. Medical students who are skillful and able to think critically will excel in the modern, complex hospital environment. This necessitates extensive CT development among medical students. Average CTDI-CV score in this study was greater than 280, signifying that Chinese medical students had an overall positive CT disposition. The result echoed recent studies conducted in China by Wang [22] and Pu [18]. In terms of subscales scores, study participants scored the highest on “inquisitiveness” and lowest on “truth-seeking.” Interestingly, this finding echoed a similar finding from a previous study, which suggested that Chinese medical students were curious and enthusiastic to seek knowledge but were less persistent about truth-seeking [11]. However, a study by Bixler revealed that medical students at the Ohio State University College of Medicine demonstrated relatively strong CT with a mean overall score of 310.7 [12]. The differences suggest that Chinese medical students’ CT dispositions are weaker than that of Western countries’ students. Similarly, research by Tiwari [23] and Yeh [24] on nursing students suggested that Chinese health professional students’ CT dispositions were inferior to those of students from Western countries. These CT score differences may be attributed to educational and cultural differences.

Considering the elements of medical education, results of this study demonstrated that Chinese medical students’ disposition for CT gradually decreased as students progress from the stage of basic science courses to the stages of clinical courses and internship. Similarly, the one-year follow-up study of 112 medical students in the clinical stage of their education confirmed that students suffered significant decline in total CT scores and the five sub-scale scores as they progressed through their education. Although the one-year follow-up mean score of CTDI-CV was still above the 280 threshold for “positive disposition,”,the percentage of medical students with a positive disposition for CT has significantly dropped. This may indicate possible significance of this decrease. These results echoed studies abroad, which illustrated that medical students’ CT may improve moderately or decline significantly while in medical school [25, 26]. As we know, three institutions in this study applied traditional discipline-centered curriculum when the investigation was implemented. Methods to improve CT included successful curriculum integration such as by reducing the overlap of different disciplines and giving students greater time to spend on autonomous learning and practicing independent thinking [8] were not implemented. What’s more, active teaching methods such as case studies [27], problem-based-learning (PBL) [28, 29], concept mapping [30], as well as formal assessments [31] were also not widely used. A recent qualitative study on barriers of CT in medical students’ curriculum reveal that this kind of traditional and unchanging system of education was a significant barrier to the implementation of the critical thinking program [32]. As a result, this study suggested that current Chinese medical education may hinder development of medical students’ CT, further highlighting the glaring need to reform.

This study also found several specific educational influencing factors on CT for medical students. The mean CT score achieved by the students at Tongji University School of Medicine was significantly higher than the mean scores achieved by students at the two other medical schools. Tongji is granted both the “985” and “211” grants and can obtain more funding and recourse from the Ministry of Education and government. Readily available funding for medical education in turn attracts more excellent students to enroll and more qualified faculty to teach. The combination of more self-motivated students and superior faculty may contribute to higher CT scores. Furthermore, due to the unsatisfying social and economic circumstances that doctors in China face, some Chinese medical students now have lower desires to become doctors [33]. However, our study found that although only 64.0% of medical students showed a strong interest in medicine, 91.5% still wished to become doctors after graduation. The attitude of students towards their occupations was positively correlated with their CT dispositions. Students who were interested in medicine may spend more time on their studies and had higher GPAs as well as higher CT scores. One explanation for this may be that students who are devoted to medicine will be more motivated to learn independently and think actively [34]. This study found that while 73.3% of students believed that CT was important, only 45.0% of students had an accurate understanding of what critical thinking actually was. These results indicate that although CT has been targeted as an important area needing improvement, it remains relatively neglected when compared to other more in vogue medical education topics such as medical knowledge, clinical practice, and the humanities. CT-related goals and its required methodology should be specified more clearly [35].

There are also cultural differences from China and western countries. China is a collectivist society and does not always encourage thinking outside the norm or questioning authority [36]. Chinese youth who challenge authority may be considered rude or disrespectful. As a result, Chinese youth may learn to avoid confrontation or open discussion with authority and avoid voicing dissenting views [37]. In general, Chinese students are discouraged from questioning teachers as teachers do not want to lose face if they do not know the answers [38]. This setting is not optimal for promoting critical thinking and demonstrates a need to create a safe, supportive, higher learning educational environment [39].

### Strengths and limitations

This study and its design had several strengths and limitations. The major strength of this cross-sectional study was its sizeable sample and its representativeness of medical students from multiple different classes of medical schools. Furthermore, a follow-up study design was conducted to verify the impact of medical education on students’ CT disposition as well. However, there were several limitations. Firstly, all data in this study were based on self-reported questionnaires. Therefore, inaccuracies caused by memory biases and subjective attitudes were unavoidable. Secondly, although the CT assessment tool of was widely used among medical students in China, it was only validated among Chinese nursing students, and CTDI-CV scores and statistical significance may not reflect students’ real-word performance or clinical significance. Thirdly, the sample size of the longitudinal study was small. CTDI-CV score was still above the 280 threshold for “positive disposition” at the end of 1 year follow-up although it was significantly decreased. As such, statistical significance may not reflect students’ real-word performance. Lastly, data from 20% of eligible participants was not included in the data analyses, which excluded potentially analyzable data.

## Conclusions

In this study, Chinese medical students generally exhibited positive CT dispositions. One-year follow-up comparison showed that the percentage of medical students with a positive disposition for CT has significantly dropped as students progressed through medical education. Academic and perceptive factors were also investigated to benefit Chinese medical educators and to encourage curricula reform. Results from this study contribute to understanding the status of Chinese medical students’ CT disposition.

## Data Availability

The datasets used and/or analysed during the current study are in Chinese and are available from the corresponding author on reasonable request but will require translation to English.

## Abbreviations

IIME: International Medical Education
GMER: Global Minimum Essential Requirements
CTDI-CV: Critical Thinking Disposition Inventory-Chinese Version
CT: Critical Thinking
CVI: Content Validity Index
PBL: Problem-based-learning
GPA: Grade point average
